# Greater perceived stress management skills and heightened brain metabolic activity in cortical and subcortical stress processing regions in metastatic breast cancer patients

**DOI:** 10.1007/s11682-023-00821-2

**Published:** 2023-11-11

**Authors:** Joaquim C. Reis, Luzia Travado, Aaron S. Heller, Francisco P. M. Oliveira, Silvia D. Almeida, Berta Sousa, Durval C. Costa, Michael H. Antoni

**Affiliations:** 1 Institute of Biophysics and Biomedical Engineering, Faculty of Sciences, University of Lisbon, Lisbon, Portugal; 2 Breast Unit, Champalimaud Clinical Center, Champalimaud Foundation, Lisbon, Portugal; 3 Department of Psychology, University of Miami, and Sylvester Comprehensive Cancer Center, Cancer Control Program, University of Miami Miller School of Medicine, Miami, FL USA; 4 Nuclear Medicine - Radiopharmacology, Champalimaud Clinical Centre, Champalimaud Foundation, Lisbon, Portugal; 5 Division of Medical Image Computing, German Cancer Research Center, Heidelberg, Germany

**Keywords:** Brain metabolism, Emotional distress, Metastatic breast cancer, Stress management skills, Neural stress processes

## Abstract

**Purpose:**

Emotional distress and adversity can contribute to negative health outcomes in women with breast cancer. Individual differences in perceived stress management skills such as cognitive reframing and relaxation for coping with adversity have been shown to predict less distress and better psychological and physiological adaptation. Prior work shows that more distressed breast cancer patients reveal less metabolic activity in brain regions such as the insula, thalamus, ventromedial and lateral prefrontal cortices. This led us to pose the hypothesis that breast cancer patients with greater stress management skills (e.g., ability to reframe stressors and use relaxation) may conversely show greater activation in these brain regions and thereby identify brain activity that may be modifiable through stress management interventions. The main objective of this study was to examine the association of perceived stress management skill efficacy with the metabolism of 9 key stress-implicated brain regions in women diagnosed with metastatic breast cancer.

**Methods:**

Sixty women (mean age 59.86 ± 10.04) with a diagnosis of mBC underwent ^18^F-fluorodeoxyglucose positron emission tomography. Perceived stress management skill efficacy was assessed with the Measure of Current Status Scale.

**Results:**

Greater perceived stress management skill efficacy related significantly to higher metabolic activity in the insula, thalamus, ventromedial and lateral prefrontal cortices, and basal ganglia; this network of regions overlaps with those previously shown to be under-activated with greater level of distress in this same sample of metastatic breast cancer patients.

**Conclusion:**

This is the first study to demonstrate in metastatic cancer patients that greater perceptions of stress management skill efficacy are associated with metabolic activity in key brain regions and paves the way for future studies tracking neural mechanisms sensitive to change following stress management interventions for this population.

**Supplementary Information:**

The online version contains supplementary material available at 10.1007/s11682-023-00821-2.

## Introduction

Emotional distress and adversity can contribute to negative health outcomes in cancer patients through stress-related biobehavioral processes that could promote cancer progression and shorter survival and compromise quality of life (Antoni et al., 2006a; Giese-Davis et al., 2011; Lutgendorf & Andersen, 2015). Individual differences in perceived stress management skills such as cognitive reframing and relaxation for coping with adversity have been shown to predict less distress and better psychological and physiological adaptation in different medical populations including cancer patients (Reis et al., 2022) and persons with other conditions such as chronic fatigue syndrome (Hall et al., 2014; Lattie et al., 2012) and prostate cancer (Penedo et al., 2013; Yanez et al., 2015). Despite evidence that these differences in perceived stress management skills are related with psychological and physiological adaptation, the neural mechanisms underlying these perceptions of stress management skills remain unknown.

Identifying specific brain regions and networks that are sensitive to fluctuations in psychological states and perceptions of stress management skills, during the cancer journey can provide insights into the “hard-wiring” of a biobehavioral model of cancer, and may guide the development of more targeted psychosocial, behavioral, and pharmacological interventions to optimize psychological adaptation, quality of life, and health outcomes in cancer survivors (Reis et al., 2020a, 2020b). For example, the sympathetic–adrenal–medullary (SAM) axis and the hypothalamic–pituitary–adrenal (HPA) axis are brain-body pathways that play a crucial role linking distress with physical health in general (Gianaros & Wager, 2015) and with cancer progression in particular (Antoni et al., 2006b). Although activation of the HPA and SAM axes are governed by subjective stress appraisals and changes in concomitant brain circuits (Godoy et al., 2018) that can mediate distress states and downstream health outcomes in treated cancer patients, little is known about neural mechanisms related to such perceptions.

We also know that CBT-based stress management interventions improve breast cancer patients’ ability to manage their stress via increased perceived stress management skills (PSMS). Theses skills, including relaxation and cognitive restructuring, relate to reductions in distress, negative mood, and objective stress responses (serum cortisol) (Antoni et al., 2006a, b, c; Phillips et al., 2011). Within a separate sample of women with metastatic breast cancer (mBC) we found that greater PSMS related to less distress, which in turn, was related to better regulated diurnal cortisol pattern (Reis et al., 2023), suggesting that this phenomenon may persist across the cancer continuum. However, we do not yet understand how the subjective sense of stress management skill is represented in the activity of specific brain regions in these patients.

Neuroimaging studies that correlate emotional distress with neural circuitry in cancer patients find changes in function and structure of key brain regions such as the prefrontal cortex (PFC), thalamus (Th), amygdala (Amy), hippocampus (Hi), anterior cingulate cortex (ACC), hypothalamus (Hy), basal ganglia (BG, striatum and caudate) and insula (Ins) (Reis et al., 2020a, 2020b). Recently, in metastatic breast cancer (mBC) patients, we reported that heightened emotional distress was significantly associated with reduced ^18^F-fluorodeoxyglucose (^18^F-FDG)-based brain metabolism in the Ins, Th, Hy, ventromedial (vm) PFC, and lateral (l) PFC (Reis et al., 2020a, 2020b). One critical question is whether the subjective sense of being able to cope with stress, measured as PSMS efficacy, might rely on these same neural circuits, resulting in a greater activation of these brain regions. Another question is whether perceived efficacy for specific stress management skills—cognitive restructuring, tension awareness, relaxation, interpersonal skills—have the greatest impact.

The cognitive neurophysiological model of CBT for anxiety and depression proposes that coping engages brain regions related to cognitive control (Clark & Beck, 2010). More specifically, this model proposes two modes of information processing: (1) an automatic, reflexive, bottom-up mode related with the hyperactivation of the amygdala, hippocampus and alteration of the vmPFC; this cognitive processing is the key to emotion generation; and (2) an effortful, symbolic, top-down mode, based on cognitive control of emotion, which involves the activation in the activity of the ACC, vmPFC, lPFC and orbitofrontal cortex (Mitchell et al., 2007; Ochsner & Gross, 2005; Wyland et al., 2003). Symptom reduction in anxiety and depression is attributed to the strengthening of the more reflective, top-down cognitive control through increased activation of these brain regions (Clark & Beck, 2010). We thus hypothesize that lower activity of these brain regions such as lPFC, vmPFC, Ins, Hy and Th, which we previously found correlated with emotional distress in mBCa patients (13), may be reversed in persons with greater stress management skills for reducing negative mood and distress (Antoni & Dhabhar, 2019; Reis et al., 2020a, 2020b). In other words, we hypothesize here that individual differences in PSMS efficacy are linked to upregulated metabolic activity in similar distress-related brain regions in women with mBC.

Our aim is to examine the associations of overall and specific (cognitive restructuring, tension awareness, relaxation, interpersonal skills) domains of PSMS with activity in theorized brain regions in mBCa patients. For instance, it remains unknown whether perceived stress management skills such as reappraisal of maladaptive thinking are associated with the activity of the same brain regions as are other skills such as relaxation. To do so we examined the association of PSMS efficacy with the ^18^F-fluorodeoxyglucose (^18^F-FDG)-based brain metabolism of 9 key stress-implicated brain regions (Ins, Th, Hy, vmPFC, lPFC, BG, Hy, ACC and Amy) in women diagnosed with mBC. We hypothesized that greater self-reported PSMS efficacy would relate to more activity of the key brain regions noted above. To further determine the specificity of these effects, we performed two additional exploratory analyses. First, we examined whether specific PSMS efficacy domains (cognitive coping skills, relaxation, tension awareness and interpersonal skills) differentially predicted metabolic activity in these key brain regions. Second, we performed an exploratory whole-brain, voxel-wise correlation between PSMS efficacy and brain metabolism, to examine if the PSMS associations are specific to the regions of interest (ROI) or if they might relate to other brain regions as well. The present study is the first to investigate the association of PSMS efficacy with activity in distress-associated neural regions and is the first to do so in women being treated for mBC.

## Methods

### Study population

Participants were women diagnosed with mBC receiving treatment at the Breast Unit of the Champalimaud Clinical Center in Lisbon, Portugal. All patients that met the following study criteria were invited to participate in the study by oncologists of the Breast Unit.

### Inclusion criteria

Study inclusion criteria were designed to create a reasonably homogeneous sample and included: 1) women > 18 years old, 2) an Eastern Cooperative Oncology Group (ECOG) performance status of 0 or^1^ (Oken et al., 1982); 3) the presence of metastatic breast cancer not amenable to curative treatment by surgery or radiotherapy; 4) having positive hormone receptors and HER2-negative disease; 5) undergoing treatment with endocrine therapy (i.e., tamoxifen or aromatase inhibitors) and/or oral chemotherapy (vinorelbine, capecitabine, or metronomic cyclophosphamide/methotrexate) or targeted therapy; 6) receipt of first-line or second-line of treatment; and 7) adequate bone marrow, coagulation, liver, and renal function (assessed by physician at the clinical visits).

### Exclusion criteria

Patients were not eligible to participate if they had any of the following: 1) the presence of brain or other CNS metastases; 2) receipt of radiation therapy to the brain or skull lesions; 3) the presence of neurodegenerative or neurologic disease; 4) current treatment with corticosteroids or intravenous chemotherapy; 5) HER2-positive breast cancer; or 6) the presence of visceral crisis, and/or significant burden of disease.

Eligible patients were informed about the study, invited to participate, and signed an informed consent form. From a total of 65 patients recruited, 5 were excluded during the study, 1 due to quick deterioration of their health condition and the others because they did not complete the questionnaires. Table 1 presents the clinical and demographic characteristics of the sample.

**Table 1:** Clinical and demographic characteristics of participants (N = 60)

	%	Mean ± SD	Mdn	Min–Max
Age (y)		58.95 ± 10.62	59	43–84
Ethnicity:				
White	100			
Marital status:				
Never married	8.3			
Married	70			
Divorced	15			
Widowed	6.7			
Stage of disease				
IV	100			
Education (y)^1^		14.98	17	9–20
Disease duration (months)		105.28	99.37	
Time from Metastasis Diagnosis to18F-FDG PET/CT scan (months)		19.16	18.15	
Measure of Current Status **(**MOCS) score		29.18	9.26	
Body Mass Index		26 ± 5.19	25.6	17.9—41.3
Plasma glucose levels		96.76 ± 17.82	92.5	70—150
Salivary cortisol indices (ng/mL)				
Waking *(4 ng/mL)		3.92 ± 0.34	3.56	0.21 -12.15
5 p.m. *(1 ng/mL)		1.37 ± 0.182	1.03	-0.34—17.2
Bedtime *(0.5 ng/mL)		1.59 ± 0.33	0.96	-0.34—17.2
Salivary IL1-β level (ng/mL)—5 p.m		242.5 ± 293.24	138.9	4.23 – 1553.97

* Normal diurnal salivary cortisol according to the supplier (Salimetrics). Compared with normal diurnal cortisol levels in healthy people, the women who participated in this study have slightly higher cortisol levels at 5 p.m. and substantially higher at bedtime. We were unable to find the normal values of IL1-β from the supplier

^1^Years of full-time education completed

### Psychotropic drugs

Because many patients in our sample were taking psychotropic drugs for anxiety and/or depressive symptoms (i.e., a serotonin-norepinephrine reuptake inhibitor [SNRI]), we performed an independent samples t-test to examine differences in metabolic activity in each ROI between patients taking antidepressants (n = 33) and those not (n = 27).

### 18F-FDG PET/CT scanning

All eligible patients underwent a positron emission tomography/computed tomography investigation with ^18^F-FDG (^18^F-FDG PET/CT) on the Philips Gemini TF16. Intravenous (IV) administration of ^18^F-FDG (227.4 ± 55.9 MBq) was always performed in the same room dimmed light and under similar conditions for all patients: eyes open, lying flat on a couch after a period of 10–15 min rest before the injection. ^18^F-FDG PET/CT scans were acquired for 15 min, starting 180 min post-injection (p.i.). LOR-RAMLA reconstruction algorithm (Philips Gemini TF16) was used for data reconstruction, with attenuation and scatter correction, using a sharp reconstruction filter, according to the routine protocol. An isotropic voxel size of 2 mm was defined. The acquisition was done at the Nuclear Medicine-Radiopharmacology Champalimaud Foundation, Lisbon, Portugal. We specifically acquired delayed ^18^F-FDG PET/CT (180-min p.i. scan) because time p.i. influences brain ^18^F-FDG uptake, increasing to a plateau and slowly decreasing, while ^18^F-FDG is exiting the cell’s compartment. The delayed acquisitions reflect much more the brain´s metabolic component than the earlier radioligand vascular distribution, resulting in improved contrast between grey and white matter (Varrone et al., 2009) compared to standard acquisitions (approximately 45 to 60-min p.i.). Previous studies, examining ^18^F-FDG uptake rates of different p.i. scans, demonstrate that ^18^F-FDG PET/CT imaging at delayed times can increase diagnostic accuracy (Wang et al., 2018). Just for sake of exemplification, compared to standard brain acquisition, a 180-min ^18^F-FDG PET/CT acquisition provides a more accurate diagnosis of large blood vessel inflammation (I. Martínez-Rodríguez et al., 2013; Isabel Martínez-Rodríguez et al., 2014).

### Imaging Processing

The 180-min ^18^F-FDG brain images were registered to the Montreal Neurological Institute (MNI) space (atlas ICBM 152 (version 2009a) Nonlinear Symmetric T1, resampled to an isotropic voxel size of 2 mm), using a suitable deformation model based on rigid, affine and deformable cubic B-spline transformations. Then the registered images were smoothed with a Gaussian kernel with 8 mm full width at half maximum and then intensity normalized. This normalization was done independently per patient, using the pons as the reference region, i.e., each voxel standardized uptake value (SUV) was divided by the mean SUV of pons, identified in this work as SUV ratio (SUVr). This ratio is a relative measure of the regional cerebral metabolic rate based on ^18^F-FDG uptake (glucose consumption). After spatial normalization to MNI space, intensity normalization and smoothing, each brain image was segmented into the 9 key brain regions characterized as regions of interest (ROIs) for this study, according to the automated anatomical labeling (AAL) brain atlas (Tzourio-Mazoyer et al., 2002). Before applying the ROI mask from the AAL brain atlas to the normalized ^18^F-FDG images, those ROIs were eroded by 2 mm in each direction to minimize the errors that might be introduced by partial volume effects and cortical thickness. Mean SUVr values were extracted for each region, per patient, for the 180-min pi image. Registration techniques were implemented in Python language, using *Pycharm* as the interpreter and *Simple Elastix* (Marstal et al., 2016) for image registration.

### Perceived Stress Management Skills Assessment

Two days after ^18^F-FDG PET/CT investigation, participants completed the Measure of Current Status** (**MOCS) scale (Carver, 2006). The MOCS is a 13-item scale (see Table 2) that measures perceived efficacy in using several stress management skills such as tension awareness, relaxation, cognitive coping skills (cognitive restructuring), and interpersonal skills such as assertiveness. The MOCS has been used in studies of women with non-metastatic breast cancer and shown to be reliable (Antoni et al., 2006a, b, c). We computed the Cronbach’s alpha test to calculate the scale reliability.

**Table 2:** Measure of Current Status **(**MOCS) scale and the 4 subscales

Subscale	Items
Relaxation	I am able to use muscle relaxation techniques to reduce any tension I experience I am able to use mental imagery to reduce any tension I experience
Awareness of tension	I become aware of any tightness in my body as soon as it develops I can easily recognize situations that make me feel stressed or upset I notice right away whenever my body is becoming tense
Interpersonal Skills	I can clearly express my needs to other people who are important to me It's easy for me to go to people in my life for help or support when I need it I can ask people in my life for support or assistance whenever I need it
Cognitive Coping Skills	I can easily stop and re-examine my thoughts to gain a new perspective It's easy for me to decide how to cope with whatever problems arise When problems arise I know how to cope with them I am confident about being able to choose the best coping responses for hard situations I can come up with emotionally balanced thoughts even during negative times

### Statistical analysis

Statistical analyses were done with IBM SPSS v27 software and a significance level of 5% (2-sided) was set. To examine the correlation between levels of PSMS efficacy (MOCS total score) and the mean SUVr in the nine ROIs, a linear regression model was applied to the 180-min p.i. acquisition brain images. In individual regression models predicting ROI SUVr, MOCS total score was the primary predictor of interest, while age was included as covariate. We included age as covariate because there is evidence that age influences brain metabolism and a meaningful decline in the SUVr in multiple brain regions has been reported as age increases (Castellano et al., 2019). SUVr, in each of the predefined ROIs, was included as the outcome variables in separate regression tests where we calculated 95% confidence intervals for standardized beta coefficients. We also performed the Benjamini–Hochberg procedure to adjust for multiple comparisons across the statistical models (Benjamini & Hochberg, 1995). For exploratory analyses we also performed: (1) partial correlations between PSMS domains (cognitive coping skills, tension awareness, interpersonal skills, and relaxation) and SUVr in the 9 ROIs, controlling for age, and (2) an exploratory whole-brain voxel-wise Pearson’s correlation between PSMS and the SUVr.

An *apriori* power analysis, using the G ∗ power 3.1 program (Erdfelder et al., 1996) and setting a linear multiple regression with a moderate effect size (f2 ​ = ​0.15) (Cohen, 1988), an alpha ​ = 0​0.05 (two-sided) and a Beta ​ = ​0.20 (power of 0.80) indicated a requisite sample size of 55 for the study.

## Results

In the present study the MOCS total score and subscale scores had good reliability (MOCS total score [α = 0.80]; tension awareness [α = 0.87], relaxation [α = 0.85], cognitive coping skills [α = 0.89], and interpersonal skills [α = 0.80]).

### Preliminary Analyses

In nine regression analyses, we included PSMS as the predictor, the SUVr of each of 9 ROIs as the outcome, and age as covariate. We included age as a covariate because, while age was not correlated with the MOCS total score (r = -0.228; p = 0.080), age was significantly negatively associated with all ROIs (r’s > -0.50; p < 0.001). The output of the independent samples t-test to examine differences in metabolic activity in each ROI between patients taking antidepressants (n = 33) and those not (n = 27) did not indicate statistically significant differences between the 2 groups in any ROIs’ SUVr (p’s > 0.05). Thus, in the regression analysis we include only age as covariate.

### Main Analyses: Perceived Stress Management Skill Efficacy and Brain Metabolic Activity in Regions of Interest (ROIs)

Table 3 presents the coefficients of the multiple linear regression analyses for PSMS efficacy (MOCS – total score), age and SUVr in the 9 ROIs. PSMS was positively associated with SUVr of the Ins, Th, vmPFC, lPFC and BG after adjusting for multiple comparisons. Age was significantly negatively correlated with SUVr in all ROIs.

**Table 3:** Multiple linear regression analyses for PSMS (MOCS – total score), and SUVr in the 9 ROIs, controlling for age in women with metastatic breast cancer (N = 60)

Region	Overall R^2^	F statistics		MOCS total score			Age	
			std β	CI^a^	p	std β	CI^a^	p
Insula	0.460	F(2, 57) = 24.257 p < 0.001	0.235	[0.034, 0.435]	0.022*	-0.585	[-0.785, -385]	< 0.001
Thalamus	0.398	F(2, 57) = 18.856 p < 0.001	0.352	[0.140, 0.563]	0.002*	-0.450	[-0.661, -0.239]	< 0.001
Hypothalamus	0.392	F(2, 57) = 18.375 p < 0.001	0.164	[-0.048, 0.377]	0.127	-0.568	[-780, -0.358]	< 0.001
Ventro-medial PFC	0.421	F(2, 57) = 20.691 p < 0.001	0.267	[0.060, 0.474]	0.013*	-0.533	[-0.741, -0.326]	< 0.001
Lateral PFC	0.384	F(2, 57) = 17.767 p < 0.001	0.300	[0.086,0.514]	0.007*	-0.478	[-0.692, -0.264]	< 0.001
Basal Ganglia	0.346	F(2, 57) = 15.059 p < 0.001	0.306	[0.086, 0.526]	0.007*	-0.437	[-0.658, -0.217]	< 0.001
Hippocampus	0.325	F(2,57) = 13.728 p < 0.001	0.087	[-0.136, 0.311]	0.437	-0.544	[-0.768, -0.320]	< 0.001
ACC	0.386	F(2,57) = 17.945 p < 0.001	0.182	[-0.032, 0.395]	0.094	-0.554	[-0.768, -0.341]	< 0.001
Amygdala	0.135	F(2,57) = 4.446 p = 0.016	0.080	[-0.173,0.334]	0.528	-0.341	[-0.594, -0.87]	0.009

^a^Bootstrap confidence intervals (95%) for standardized beta coefficient, using 5000 bootstrap samples

^*^Significant p-values after Benjamini–Hochberg adjustment (alpha = 0.05)

### Association of Perceived Stress Management Skill Efficacy Subscales with Brain Metabolism in ROIs

To explore possible links between PSMS efficacy in specific domains and neural metabolism of the 9 ROIs, we performed partial correlations between MOCS subscales (cognitive coping skills, awareness of tension, interpersonal skills and relaxation) and the 9 ROIs, controlling for the effects of age. Since these are exploratory analysis, we did not make adjustment for multiple comparisons. As presented in Table 4, the cognitive coping skills subscale was the one that was most broadly linked to the ROIs, including the Ins, Th, vmPFC, lPFC, BG, Hy, and ACC. The awareness of tension subscale was significantly associated with metabolism in the Th and BG. The interpersonal skills subscale did not correlate with any ROI. Lastly, the relaxation subscale was significantly associated with Th activity only.

**Table 4:** Partial correlations between MOCS subscales and SUVr for the 9 ROIs, controlling for age in patients with metastatic breast cancer (N = 60)

	Cognitive coping skills	Awareness of tension	Interpersonal skills	Relaxation
	Correlation *p* BootCI^a^	Correlation *p* BootCI^a^	Correlation *p* BootCI^a^	Correlation *p* BootCI^a^
Insula	0.295	0.240	0.115	0.190
	0.023*	0.067	0.387	0.150
	[0.059,0.533]	[-0.003,0.004]	[-0.115,0.347]	[-0.040,0.448]
Thalamus	0.413	0.344	0.056	0.292
	0.001*	0.008*	0.675	0.025*
	[0.175,0.617]	[0.113,0.561]	[-0.163,0.268]	[0.050, 0.513]
vmPFC	0.387	0.214	0.121	0.166
	0.002*	0.103	0.362	0.209
	[0.170,0.570]	[-0.006,0.422]	[-0.147,0.361]	[-0.068, 0.392]
lPFC	0.392	0.242	0.140	0.218
	0.002*	0.065	0.290	0.097
	[0.168,0.585]	[0.007,0.481]	[-0.123,0.404]	[-0.014,0.436]
Basal Ganglia	0.375	0.336	0.016	0.190
	0.003*	0.009*	0.903	0.150
	0.134,0.593]	[0.088,0.560]	[-0.276,0.317]	[-0.041,0.416]
Hypothalamus	0.272	0.102	-0.162	0.162
	0.037*	0441	0.422	0.220
	[0.003,0.480]	[-0.158,0.327	[-0.368,0.164]	[-0.108,0.387]
Hippocampus	0.127	0.148	0.049	-0.047
	0.338	0.265	0.713	0.726
	[-0.085, 0.331]	[-0.067,0.343]	[-0.208,0.300]	[-0.260,0.149]
Anterior cingulate cortex	0.286	0.149	0.058	0.107
	0.028*	0.259	0.664	0.420
	[0.080, 0.464]	[-0.051, 0.353]	[-0.205,0.312]	[-0.125,0.331]
Amygdala	0.146	0.047	0.033	-0.057
	0.271	0.723	0.670	0.670
	[-0.089,0.338]	[-0.129,0.215]	[-0.223,0.297]	[-0.304,0.151]

^a^ Bootstrap confidence intervals (95%) for correlation coefficient, using 5000 bootstrap samples

* Significant p values (< 0.05)

### Exploratory Whole-Brain Voxel-wise Correlation Distribution between Perceived Stress Management Skill Efficacy and Brain Activity

**Fig. 1:**
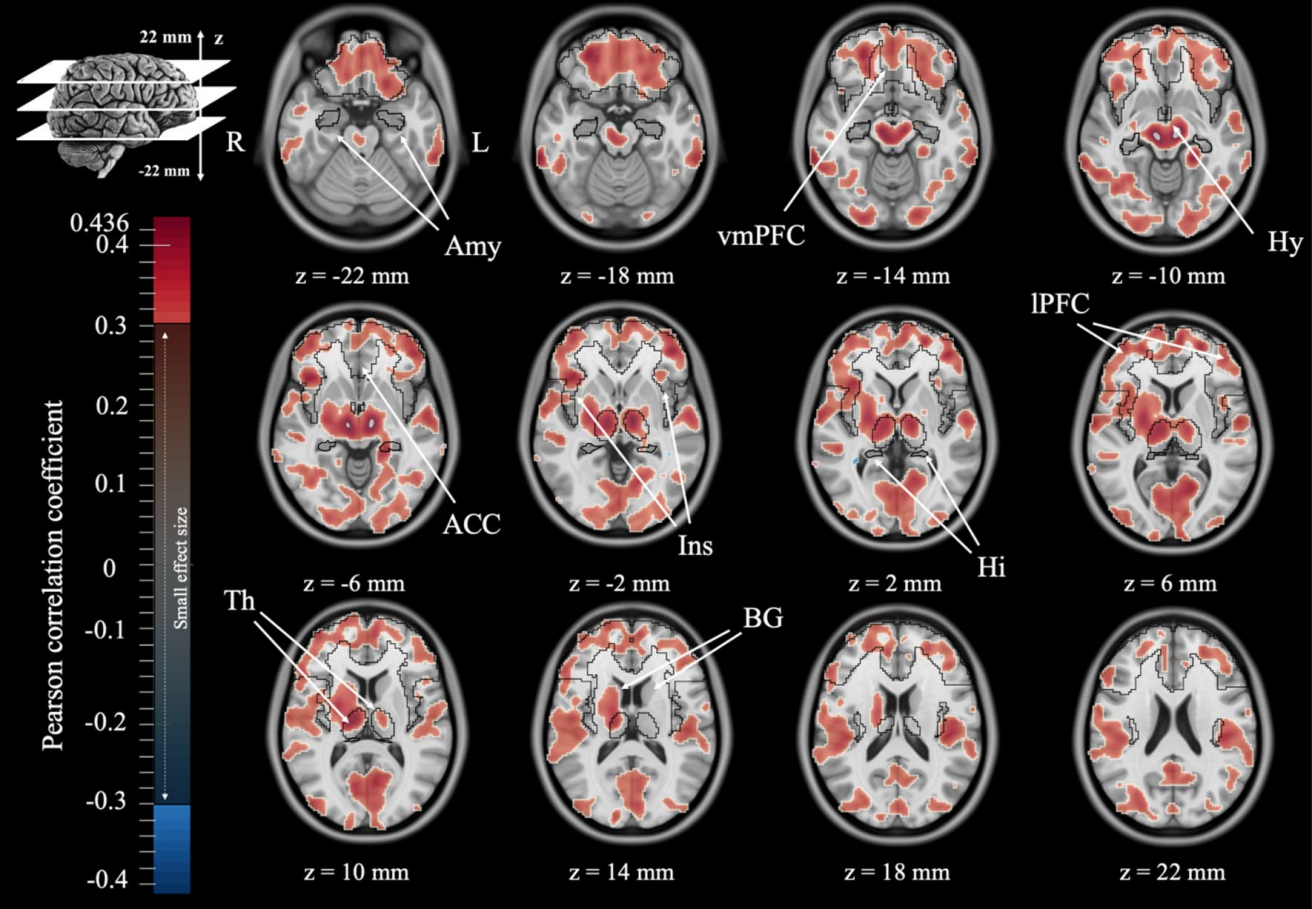
Whole-brain, voxel-wise correlation between perceived stress management skills and the standardized uptake value ratio (SUVr) is illustrated. Images represent 12 representative axial views of the Montreal Neurological Institute grayscale T1 magnetic resonance images overlapped by the Pearson correlation coefficients (color bar). Only voxels with at least a moderate Cohen effect size (r > 0.3) are represented (Cohen, 1988). Arrows indicate the 9 regions of interest (ROIs): ventromedial prefrontal cortex (vmPFC), hypothalamus (Hy), thalamus (Th), lateral prefrontal cortex (lPFC), insula (Ins), basal ganglia (BG), hippocampus (Hi), anterior cingulate cortex (ACC) and amygdala (Amy)

Figure 1 illustrates the whole-brain, voxel-wise correlation between PSMS total scores and the standardized uptake value ratio (SUVr). Broadly, there are significant associations between PSMS and the neural activity in brain regions other than the focal ROIs. Moreover, there is spatial specificity of associations between PSMS and frontal ROIs including the Ins, ACC and lPFC.

## Discussion

Women with metastatic breast cancer endure several acute and chronic stressors (Burnet & Robinson, 2000; Warren, 2010). The ability to manage these stressors has been shown to affect psychological, neuroendocrine and immune adaptation processes that could contribute to optimal disease outcomes in cancer patients (Antoni et al., 2023). However, the neural activities that orchestrate these stress response processes in patients with metastatic disease are relatively unknown. We examined for the first time associations between efficacy for using stress management skills and activity in specific brain regions in women with metastatic breast cancer. We provide evidence that higher reported perceptions of stress management skill (PSMS) efficacy relate significantly to more metabolic activity in the insula, thalamus, ventromedial and lateral prefrontal cortices, and basal ganglia. Of all these regions, the insula, thalamus, ventromedial and lateral prefrontal cortices regions have previously shown to show less activation in mBC patients reporting greater emotional distress (Reis et al., 2020a, 2020b). Further analysis indicated that the association of PSMS with a priori ROIs were mostly specific to the cognitive coping subdomain of PSMS. Higher scores on the subscale of cognitive coping skills, which assesses reappraisal of maladaptive thinking, was significantly associated with more metabolism in seven of the ROIs: insula, thalamus, ventromedial and lateral prefrontal cortices, hypothalamus, basal ganglia, and anterior cingulate cortex.

It is interesting to note a parallel with the neurophysiological model of CBT (Clark & Beck, 2010) which holds that the anterior cingulate cortex, and the lPFC and vmPFC are activated by stress management skills based on cognitive control like cognitive restructuring and cognitive reappraisal. A recent meta-analysis of studies involving non-cancer patients with affective disorders confirms that CBT-based psychotherapy activates the pre-frontal cortex (Nord et al., 2021). These data are in line with the findings of the present study which indicated that higher PSMS – often a target of CBT – was associated with more activity of the PFC, namely the ventromedial and lateral prefrontal cortices.

Prior work has shown that PSMS efficacy for cognitive restructuring is associated with lower objective indicators of stress, including afternoon-evening serum cortisol, in women with non-metastatic breast cancer (Phillips et al., 2011). In line with this, within a sample of women with metastatic breast cancer (mBC) we found that specific PSMS most strongly associated with less distress levels were those reflecting cognitive restructuring, which in turn, were related to better regulated diurnal salivary cortisol patterns (Reis et al., 2023). In the current study, PSMS reflecting cognitive restructuring was also significantly associated with more metabolic activity of hypothalamus which may indicate that the association of emotional distress with cortisol might be mediated by the metabolism of this brain region that is crucial for HPA axis functioning. We correlated MOCS score with cortisol levels (see supplemental material) and the results indicated that higher PSMS efficacy is significantly related with smaller cortisol slope values, suggesting that higher PSMS is related with a healthier cortisol slope (Sephton et al., 2000).

In other words, having a greater sense of efficacy in using CBT-based stress management skills may contribute to lower self-reported distress and better regulated diurnal salivary cortisol, in part due to greater activation in the same brain regions. Among women with breast cancer, stress management skills taught in CBT-based interventions can reduce perceived threats and feelings of vulnerability by increasing confidence (self-efficacy) in using relaxation, cognitive restructuring, and coping skills to cope with ongoing stressors during treatment (Antoni, 2003). Identifying individual differences in the perceived efficacy for using these skills may be particularly relevant in patients with advanced cancer, who are dealing with multiple challenges of cancer and its treatment in an atmosphere of heightened uncertainty and grief (Burnet & Robinson, 2000; Warren, 2010). Patients reporting lower PSMS would be prime candidates for interventions such a cognitive behavioral stress management, which while widely tested in cancer patients with early disease has yet to be tested in those with metastatic disease (Antoni et al., 2023). The present study suggests that these patients could be monitored for contemporaneous changes in PSMS, activity in identified brain regions, and peripheral physiologic stress markers to understand the possible health effects of successful stress management.

The voxel-wise correlation distribution between PSMS efficacy and the metabolism in the whole brain indicated significant PSMS correlations with activity in other brain regions beyond our hypothesized ROIs. However, these were only exploratory analysis. The possibility of significant correlations of PSMS with many other brain regions is in line with neuroscience theories that emphasize domain-general brain networks for the processing of emotions and that specific regions should be studied in the context of brain networks and anatomical or functional connectivity patterns (Lindquist et al., 2016; Oosterwijk et al., 2012; Pessoa, 2014). Future studies in this area should not be limited to these ROIs and must include other brain regions and anatomical and functional connectivity patterns.

Strengths. Strengths of this study include the recruitment of a homogenous sample of 60 women with a specific type and stage of cancer who had a pre-defined exposure to different forms of cancer treatments, excluding current treatment with corticosteroids or intravenous chemotherapy. Thus, we minimized the potential impact of disease and treatment confounding variables that have plagued this line of research with cancer patients. For instance, prior studies relating distress to brain imaging in cancer patients were characterized by great clinical heterogeneity, composed of multiple groups, each having different types and stages of cancer and, consequently, distinct types of treatment (Reis et al., 2020a, 2020b). Also, the sample size that we used in this study is larger than that used in most of those prior studies (median N = 35) (Reis et al., 2020a, 2020b). Therefore, we have confidence that the present work provides novel evidence for associations between patient perceptions and activity in important brain regions relevant to stress processing.

Limitations. The use of a cross-sectional design and a convenience sample of patients who were lacking diversity in race/ethnicity and sociodemographic status. These factors act to limit any causal conclusions and generalizability to other populations beyond well-educated Caucasian patients with mBC. More importantly, due to the cross-sectional design it is possible that brain activity changes precede perceptions of stress management skill efficacy. Definitive evidence for a casual effect of stress management processes on changes in the brain regions identified in this study must await randomized controlled trials (RCTs) that experimentally train stress management skills and observe subsequent changes in brain metabolic activity in women with mBC compared to control conditions.

## Conclusions

This is the first study to demonstrate in cancer patients (women with metastatic breast cancer [mBC]) that greater perceptions of stress management skill efficacy are associated with more metabolic activity in key brain regions hypothesized to be involved in stress responding. This suggests that interventions providing training in CBT-based stress management techniques developed for breast cancer patients (Antoni et al., 2023) may help to improve psychological status in this population while activating key brain regions necessary for optimal stress management, which could be tested in future interventional designs. Only RCTs will reveal whether CBT-based stress management interventions can improve psychological status and possibly quality of life/well-being, in individuals with mBC through changes in brain functioning in the regions identified in this study.

### Supplementary Information

Below is the link to the electronic supplementary material.

Supplementary file1 (DOCX 17 KB)

## Data Availability

The datasets analyzed in this study are available from the corresponding author upon reasonable request.

## Footnotes

1 The ECOG scale is used to assess performance status and for prognosis assessment and ability to tolerate chemotherapy; an ECOG score of 0 indicates fully active, no performance restrictions; ECOG 1, strenuous physical activity restricted, fully ambulatory and able to perform light work; ECOG 2, capable of all self-care but unable to perform any work activities up to approximately > 50% of waking hours.
