# Supplementary material for: Greater perceived stress management skills and heightened brain metabolic activity in cortical and subcortical stress processing regions in metastatic breast cancer patients
Source: Brain Imaging Behav. 2023 Nov 11;18(1):130–40. doi: 10.1007/s11682-023-00821-2 (PMC10844387; doi:10.1007/s11682-023-00821-2)
Supplement: Supplementary file 1 — Supplementary file1 (DOCX 17 KB) [file 11682_2023_821_MOESM1_ESM.docx]

**Supplemental material**

Here we present the results of the correlation between MOCS score and salivary cortisol levels. Diurnal salivary cortisol profiles were assessed over three consecutive days at three time points (waking, 5 p.m. and bedtime). The mean of each of the individual sample collections averaged over 3 days indicated the mean levels of cortisol in each time of the day (waking, 5 p.m. and bedtime). The cortisol slope was calculated by regressing the cortisol on the 3 days average of the raw values on the time of the day. This slope is an estimate of diurnal cortisol variability for each patient as is indicated by the β value in the regression. In general, a healthy cortisol slope is negative whereas a more positive slope is believed to reflect greater hypothalamic-pituitary-adrenal dysregulation (Sephton et al., 2000). Steeper negative slopes are represented by smaller values, indicating cortisol declining more rapidly during the day. Larger values represent flatter slopes, indicating slower declines, abnormally timed peaks or increasing levels during the day.

Table S1. Pearson correlations between MOCS total score and diurnal salivary cortisol indices (N = 60). Significance level of 5% (two-sided).

|  | **Cortisol waking**  Correlation  *p*  CI^a^ | **Cortisol 5 p.m.**  Correlation  *p*  CI^a^ | **Cortisol bedtime**  Correlation  *p*  CI^a^ | **Cortisol slope**  Correlation  *p*  CI^a^ |
| --- | --- | --- | --- | --- |
| **MOCS total** | 0.095  0.470  [-0.163, 0.341] | -0.124  0.347  [-0.366, 0.135] | -0.207  0.112  [-0.438, 0.049] | -0.273  0.035*  [-0.493, -0.020] |

^a^confidence intervals (95%) for correlation coefficient

* Significant p values (< 0.05)

The correlation between MOCS total score and cortisol slope indicates that higher reported perceptions of stress management skill (PSMS) efficacy relate significantly with smaller values of cortisol slope, suggesting that higher PSMS is related with a healthy cortisol slope.
